# Formation of contractile 3D bovine muscle tissue for construction of millimetre-thick cultured steak

**DOI:** 10.1038/s41538-021-00090-7

**Published:** 2021-03-02

**Authors:** Mai Furuhashi, Yuya Morimoto, Ai Shima, Futoshi Nakamura, Hiroshi Ishikawa, Shoji Takeuchi

**Affiliations:** 1 Institute of Industrial Science, The University of Tokyo, Tokyo, Japan; 2 Global Innovation Center, Nissin Foods Holdings Co., Ltd, Tokyo, Japan; 3 Department of Mechano-Informatics, Graduate School of Information Science and Technology, The University of Tokyo, Tokyo, Japan; 4 Laboratory of Clinical Regenerative Medicine, Department of Neurosurgery, Faculty of Medicine, University of Tsukuba, Ibaraki, Japan; 5 International Research Center for Neurointelligence (WPI-IRCN), The University of Tokyo Institutes for Advanced Study (UTIAS), the University of Tokyo, Tokyo, Japan

**Keywords:** Engineering, Tissues, Biological techniques

## Abstract

Owing to the increase in the global demand of meat, cultured meat technology is being developed to circumvent a shortage of meat in the future. However, methods for construction of millimetre-thick bovine muscle tissues with highly aligned myotubes have not yet been established. Here, we propose a culture method for constructing 3D-cultured bovine muscle tissue containing myotubes aligned along its long-axial direction, which contracted in response to electrical stimulation. First, we optimised the composition of biomaterials used in the construction and the electrical stimulation applied to the tissue during culture. Subsequently, we fabricated millimetre-thick bovine muscle tissues containing highly aligned myotubes by accumulating bovine myoblast-laden hydrogel modules. The microbial content of the bovine muscle tissue cultured for 14 days was below the detection limit, indicating that the muscle tissues were sterile, unlike commercial meat. Therefore, the proposed construction method for bovine muscle tissues will be useful for the production of clean cultured steak meat simulating real meat.

## Introduction

The global consumption of meat is increasing with population growth, leading to concerns of a future protein crisis.^1,2^ As the conventional livestock industries have been problematic for sustainability due to the ethical problems and its adverse effects on the environment,^3^ more sustainable technologies for meat production are required to bridge the demand-supply gap and thwart the protein crisis.^4,5^ Cultured meat constructed via tissue culture of animal cells is one of the promising candidates as sustainable next-generation meat, as it can be generated using small amounts of cells obtained without killing livestock, as well as lower land use and water footprint.^6^

Cultured meat has been constructed using 3D culture methods developed in the fields of regenerative medicine and drug discovery. For the fabrication of cultured meat from bovine myocytes, prior studies have proposed culturing of hydrogel with myocytes around a column of agarose for the formation of ring-shaped tissue,^7^ and culturing of myocyte-laden hydrogel bridged between anchors for formation of fibre-shaped tissue.^8^ As the bovine cultured muscle tissues constructed using these methods are small in size, these tissues can be used as cultured minced meat.

In addition to cultured minced meat, large cultured meat that can be used as steak is required to satisfy global dietary diversity.^7,9^ For the construction of cultured steak meat with a realistic texture, large muscle tissue with densely accumulated and unidirectionally aligned matured myotubes is required.^10^ Block-shaped tissue formation arising from culturing of myocytes in a porous gelatin or a porous soy protein scaffold has been proposed for the construction of large bovine muscle tissue.^11,12^ However, these methods result in isotropic distribution of myotubes, low density and loss of contractility; hence, methods for construction of bovine muscle tissues that can be used as steak meat are still challenging. Therefore, the culture conditions for large bovine muscle tissue must be improved to recreate the characteristics of steak meat.

**Fig. 1:**
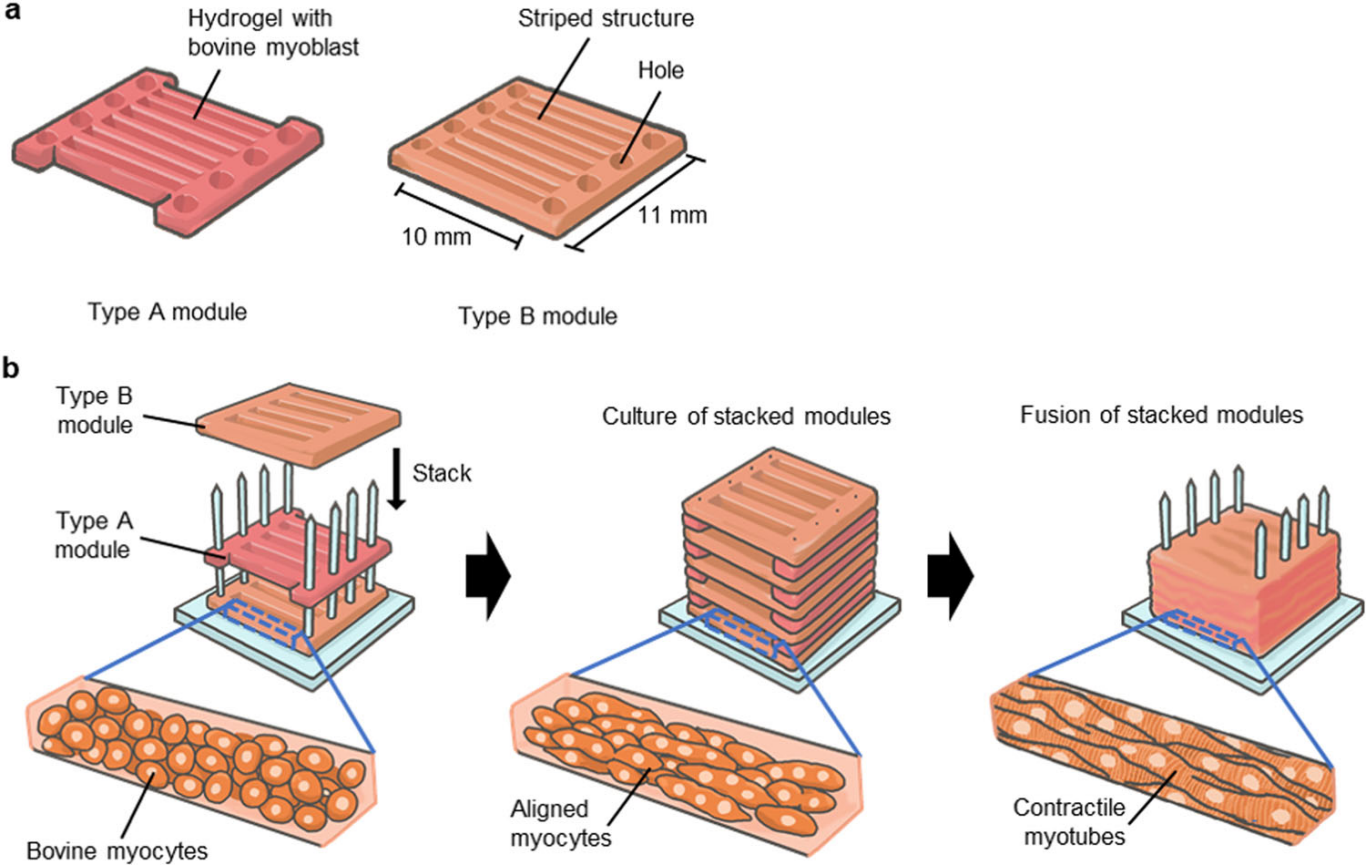
Construction process of millimetre-thick bovine muscle tissue. **a** Illustration of two types of myocyte-laden hydrogel modules composed of holes to allow immobilisation of the pillar and seven or eight striped structures to promote alignment of myocytes. **b** Conceptual illustration of the fabrication process for a millimetre-thick bovine muscle tissue.

In this study, we develop a culture method for constructing 3D-cultured bovine muscle tissue composed of unidirectionally aligned myotubes sufficiently mature to show contractility. To establish the construction method, we clarify the composition of the hydrogel covering the myocytes during culture and the effects of applying electrical stimulation to the tissue. We also develop a method for fabricating millimetre-thick bovine muscle tissues containing highly aligned myotubes using bovine myoblast-laden hydrogel modules with striped structures (Fig. 1). Here, we investigate the morphology and contractility of the bovine muscle tissue as a function of the composition of the used hydrogel and electrical stimulation. Furthermore, we evaluate the morphology and characteristics of the millimetre-thick bovine muscle tissue as food.

## Results

### Culture condition for construction of contractile bovine muscle tissue

To construct contractile bovine muscle tissue, we cultured bovine myocyte-laden hydrogel on a culture device consisting of anchors with pillars fabricated using stereolithography and a substrate consisting of polydimethylsiloxane (PDMS) (Supplementary Fig. 1). After the anchors were integrated with the substrate, the pillars were arranged to protrude from the surface of the substrate. Bovine myocytes used for the tissue construction were prepared by culturing cells collected from commercial fresh beef. To evaluate proportion of myocytes in the collected cells, we carried out flow cytometry and found that >85% cells were myocytes (myoblasts and satellite cells) (Supplementary Fig. 2). The pillars were embedded in the hydrogel by gelling the hydrogel solution containing the bovine myocytes on the substrate. When the myocytes were cultured in the hydrogel for 14 days, fibre-shaped bovine muscle tissue of diameter 295 ± 105 µm (mean ± s.d., *n* = 32) was generated, the ends of which were immobilised with pillars, showing that the length of the muscle tissue was equal to the gap between the anchors (7 mm).

**Fig. 2:**
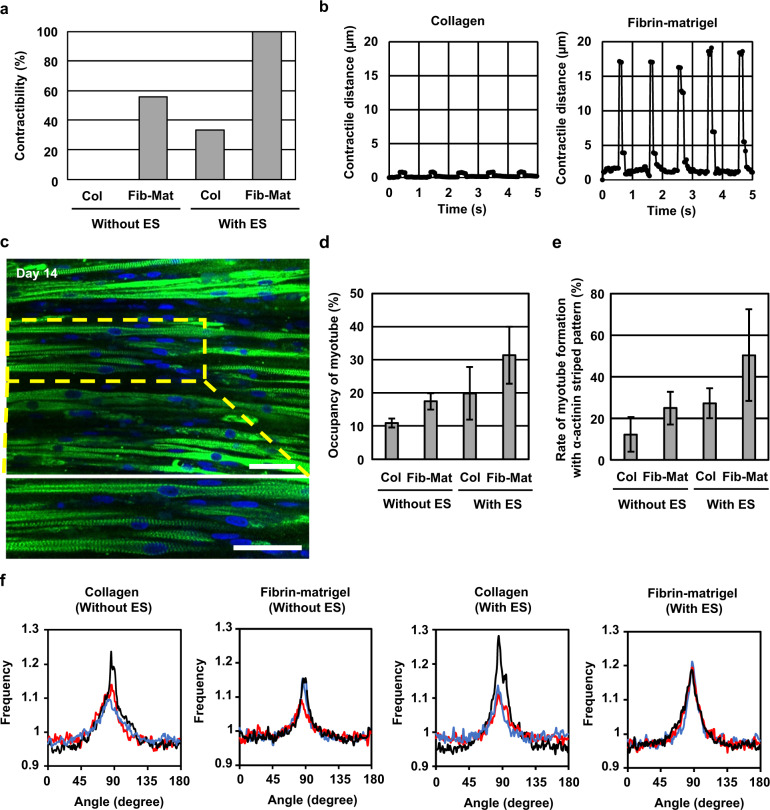
Morphological and functional analysis of bovine muscle tissue. **a** Rate of fibre-shaped bovine muscle tissues capable of contracting in response to applied electrical stimulation (the number of contractile tissue/the number of all tissues), formed with the collagen (Col)-based tissues cultured with and without electrical stimulation (ES) (amplitude: 3 V/mm, frequency: 1 Hz, duration: 2 ms) (*N* = 6), and the fibrin-matrigel (Fib-Mat)-based tissues cultured with and without ES (*N* = 9). **b** Temporal variation of contractile distance of the fibre-shaped bovine muscle tissue formed with collagen and Fib-Mat depending on the ES. **c** Confocal images of the bovine muscle tissue; cell nucleus (blue), α-actinin (green). **d** Occupancy of myotubes in the short-axial cross-section of the muscle tissue under different culture conditions (*n* = 3). **e** Rate of myotube formation with α-actinin striped patterns in all myotubes in the muscle tissue. (*n* = 3) **f** Directional distribution of brightness calculated from FFT images corresponding to confocal images. 0 degree and 180 degree show short-axial direction of the tissue and 90 degree shows its long-axial direction. Black, red and blue lines in each plot represent values for different tissues. All error bars show standard deviation. Scale bar, (**c**) 50 μm.

To compare the contractility of the cultured bovine muscle tissue prepared using different types of hydrogel, we used collagen and a mixture of fibrin and matrigel for tissue fabrication. In addition, to determine the effects of electrical stimulation on muscle tissue during culture, we stimulated the muscle tissue with electrical pulses (frequency: 1 Hz, duration: 2 ms, electrical field: 3 V/mm) for 2 h per day from day 3 to day 14; the muscle tissue was also cultured without the electrical pulses. As the result, we found that in the case of bovine muscle tissue formed using the fibrin-matrigel mixture, 100% muscle tissue stimulated with the electrical pulses (*N* = 9) and 56% muscle tissue cultured without the electrical pulses (*N* = 9) contracted according to the applied electrical pulses. In contrast, in the tissue formed with collagen, only 33% muscle tissue stimulated with the electrical pulses (*N* = 6) contracted according to the electrical pulses, whereas muscle tissue cultured without the electrical pulses (*N* = 6) did not contract (Fig. 2a). Furthermore, we confirmed that the contractile distance of the fibrin-matrigel-based muscle tissue cultured with the electrical stimulation was larger than that of the collagen-based muscle tissue (Fig. 2b) (Supplementary videos 1, 2). These results indicate that the use of fibrin-matrigel, combined with culture under electrical stimulation, facilitates construction of contractile bovine muscle tissue.

We performed α-actinin immunostaining to evaluate the maturation of bovine myotubes differentiated from the bovine myocytes in muscle tissue. The confocal images of the immunostained muscle tissue formed by culturing myocytes in fibrin-matrigel structure with electrical stimulation showed that multiple myotubes possessed striped patterns of α-actinin, indicating formation of sarcomeres in the myotubes (Fig. 2c). Using confocal imaging, we investigated the occupancy rate of myotubes and rate of formation of myotubes containing stripe patterned α-actinin in the bovine muscle tissues. The short-axis sectional images confirmed that the occupancy rates of myotubes were 11% and 17% in muscle tissues constructed using collagen and fibrin-matrigel, respectively. The occupancy rates increased by approximately twofold after culturing myocytes with electrical stimulation; 20% and 31% of the muscle tissues were constructed with collagen and fibrin-matrigel, respectively (Fig. 2d). In addition, the fibrin-matrigel-based muscle tissue cultured with electrical stimulation showed the highest rate (50%) of myotubes containing stripe patterned α-actinin among the experimental group (Fig. 2e). These results indicate that the rate of myotube formation improves when culturing myocytes in fibrin-Matrigel with electrical stimulation. Furthermore, the confocal images of the immunostained muscle tissue showed alignment of myotubes in the tissue. To quantitatively assess the orientation of the myotubes, we calculated directional distribution of the myotubes from fast Fourier transform (FFT) images based on the immunostaining images (Fig. 2f). This result indicates that the myotubes were aligned to the long axis of the tissue irrespective of the hydrogel composition and with or without electrical stimulation. The morphological evaluations also indicate that the combination of culture in fibrin-Matrigel with electrical stimulation promoted the maturation of bovine muscle tissue containing aligned myotubes.

### Construction of millimetre-thick bovine muscle tissue

**Fig. 3:**
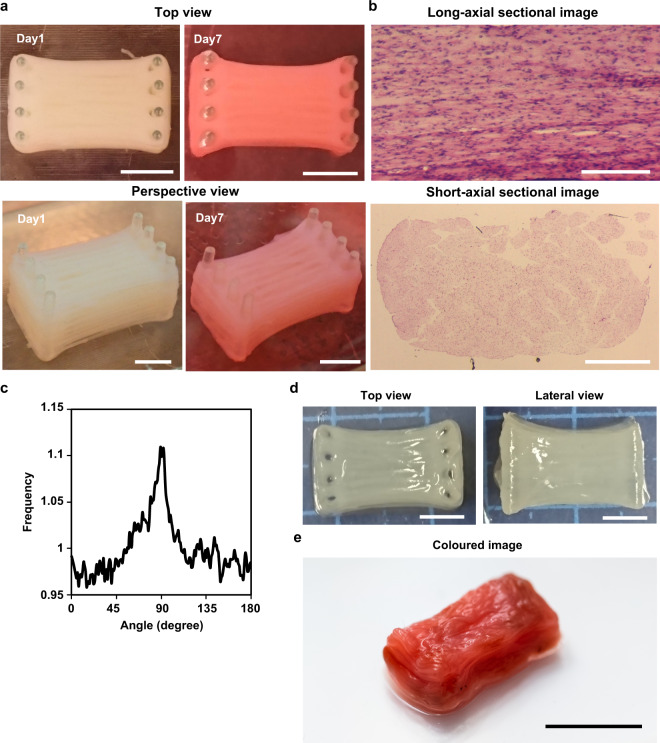
Morphological analysis of the millimetre-thick bovine muscle tissue. **a** Images of the millimetre-thick bovine muscle tissue on day 1 and day 7 of culture. The change in colour from Day 1 to Day 7 could be caused by the colour change of the phenol red in the culture medium or difference of the light exposure conditions when taking the photos. **b** Long-axial sectional image and short-axial sectional image of the muscle tissue stained with haematoxylin and eosin (H&E). **c** Directional distribution of brightness calculated from FFT images that corresponded to confocal images taken from a bovine muscle tissue constructed by stacking five myocyte-laden hydrogel modules. 0 degree and 180 degree show short-axial direction of the tissue, and 90 degree shows its long-axial direction. **d** Top and lateral views of the millimetre-thick bovine muscle tissue after release from the pillars. **e** The millimetre-thick bovine muscle tissue coloured using red food colouring agent. Scale bars, (**a**) 5 mm, (**b**) 0.2 mm (long-axial sectional image), 2 mm (short-axial sectional image), (**d**) 5 mm and (**e**) 1 cm.

For the construction of millimetre-thick bovine muscle tissue with highly aligned myotubes, we assembled myocyte-laden modules prepared using a modified version of previous methods.^13,14^ In this method, we prepared bovine myocyte-laden collagen modules shaped with a PDMS stamp. Upon culturing 40 assembled modules, the ends of which were immobilised to pillars, the modules containing 1-mm wide striped structures arranged parallel to each other at 0.3 mm intervals fused into one muscle tissue after 7 days of culture, resulting in the construction of millimetre-thick bovine muscle tissue of 8 mm × 10 mm × 7 mm (width×length×height) dimensions (Fig. 3a). To confirm integration of the striped structures in the millimetre-thick tissue, we prepared sectional images of the cultured muscle tissue stained with haematoxylin and eosin (H&E) (Fig. 3b). The short-axial sectional images showed that the myotubes were uniformly distributed, and that most of the striped structures were integrated into a single tissue. In the case where modules with 1-mm wide striped structures were arranged at 1 mm intervals following the width-height ratio (1:1) mentioned in previous studies on rat myoblasts,^13^ millimetre-thick muscle tissue was not formed, as the striped structures did not contact each other (Supplementary Fig. 3). This showed that the widths of the intervals should be smaller than the widths of the stripe structures for construction of millimetre-thick bovine muscle tissue.

We assessed the orientation of the myotubes in the millimetre-thick bovine muscle tissue from the H&E staining images. The long axis sectional images showed that myotubes were aligned to the longitudinal direction of the muscle tissue. (Fig. 3b). For quantitative evaluation of myotube orientation in muscle tissue, we prepared a cultured bovine muscle tissue consisting of five modules to visualise α-actinin using immunostaining, as observation inside immunostained millimetre-thick muscle tissue is difficult due to poor light transmission. Directional distribution of myotubes in the muscle tissue also showed that myotubes were oriented to the longitudinal direction of the tissue (Fig. 3c). From these results, we believe that the myocyte-laden module assembly method is useful for the construction of large bovine muscle tissue containing highly aligned myotubes. In addition, from the short-axial sectional image, enucleated cells were not observed, suggesting the maintenance of cellular viability without central necrosis in the tissue.

Furthermore, we assessed the feasibility of using the millimetre-thick bovine muscle tissue as cultured steak meat. Although the constructed muscle tissue was immobilised with pillars, we observed that the muscle tissue was easily released from the pillars using tweezers (Fig. 3d). Even after the muscle tissue was released from the pillars, the striped structures were maintained in the integrated state without being separated. In addition, after colouring the muscle tissue using red food colouring agent, we successfully obtained millimetre-thick bovine muscle tissue showing real meat-like appearance (Fig. 3e) (Supplementary video 3).

### Characterisation of millimetre-thick bovine muscle tissue as food

**Fig. 4:**
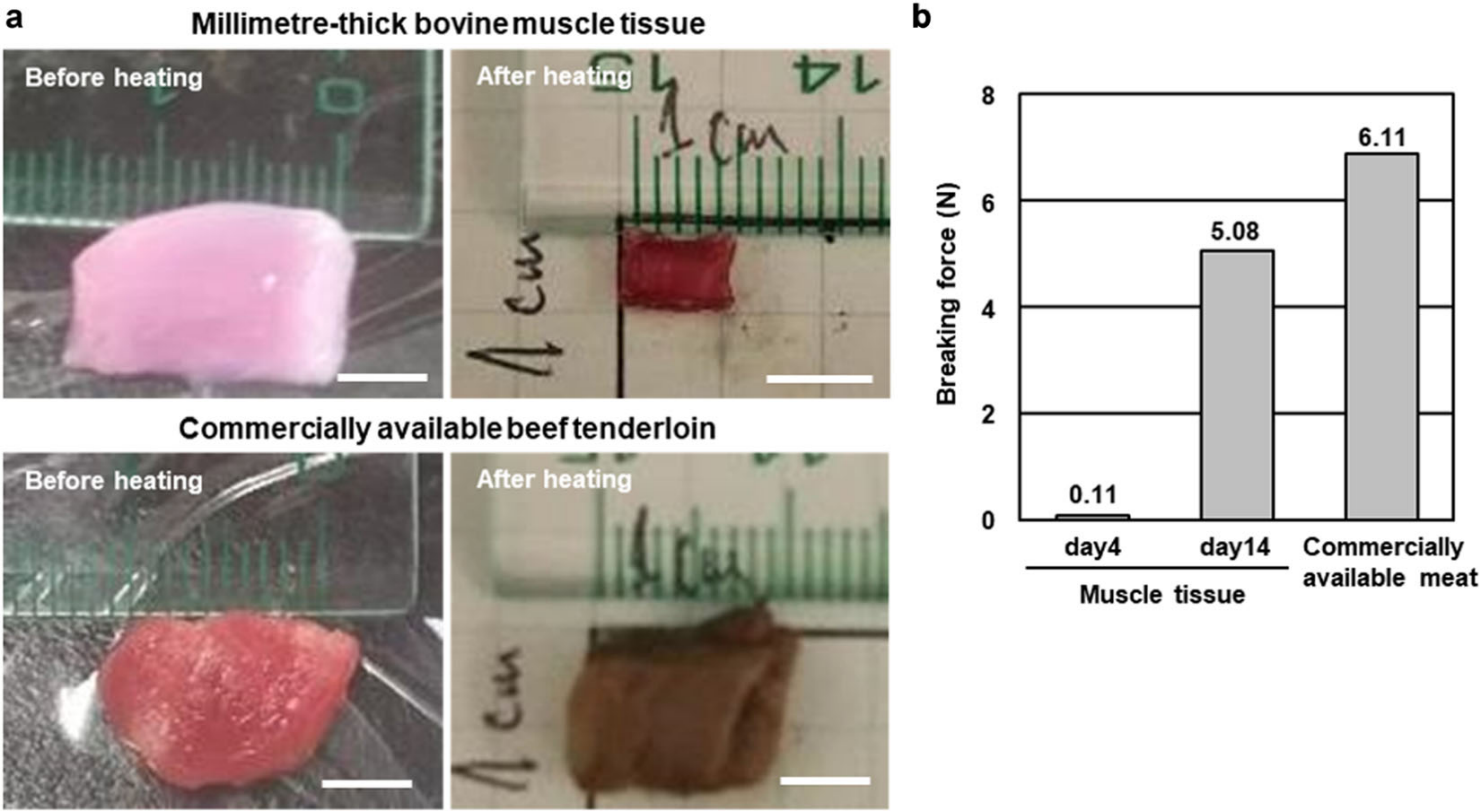
Food feature analysis of the large bovine muscle tissue. **a** Images of the millimetre-thick bovine muscle tissue and commercially available bovine beef tenderloin before and after heating (70 °C, 1 h). **b** Load at break of the muscle tissue and commercially available bovine beef tenderloin (*n* = 1). Scale bars, (**a**) 0.5 cm.

To assess the difference in texture between the cultured millimetre-thick bovine muscle tissue and commercially available steak, we measured their breaking force as an index of stiffness for food.^15^ The cultured tissue and commercially available beef tenderloin were placed in a warm bath at 70 °C for 1 h for preparing experimental samples for the measurement (Fig. 4a). The weight loss of the commercial beef tenderloin after heating was 40% (weight before heating: 0.52 g, weight after heating: 0.31 g), whereas that of the cultured tissue was 90% (weight before heating: 0.31 g, weight after heating: 0.03 g), indicating that the cultured tissue contained considerable amounts of water. In contrast, the collagen structures without any myocytes were completely melted during the heating procedure, suggesting that interactions of myocytes with collagen could change thermal responsiveness of collagen. Furthermore, we measured the breaking force using the heated samples. The breaking force of the cultured tissue on day 14 was closer to that of the beef tenderloin than to that of the cultured tissue on day 4 (Fig. 4b). This result showed that the cultured millimetre-thick bovine muscle tissue hardened with culturing, indicating that the morphological changes accompanying the culture affected the stiffness of the cultured tissue.

One of the advantages of the millimetre-thick bovine muscle tissue as cultured steak meat over commercial meat is its sterility, owing to control over the culture environment.^16^ We evaluated the microbial contamination of the muscle tissue to confirm this. Results of the general viable bacteria test showed that the amount of general bacteria in the muscle tissue cultured for 14 days was below the detection limit (<5 cfu/g), whereas that in the beef tenderloin was 1.7 × 10^5^ cfu/g. This result clearly shows that the cultured mm-thick bovine muscle tissue was cleaner than general meat in terms of microbial contamination.

## Discussion

In this study, we determined the appropriate culture conditions for constructing contractile bovine muscle tissue and showed that assembly of myocyte-laden hydrogel modules allow formation of millimetre-thick bovine muscle tissue with highly aligned myotubes along the long axis of the tissue. Evaluation of food characteristics revealed that the bovine muscle tissue showed increase in stiffness with culture and extremely low microbial contamination. As the bovine muscle tissue possesses appropriate physiological properties and food characteristics of meat, we believe that the proposed method for construction of bovine muscle tissues will be useful for producing cultured steak meat on a large scale.

Although optimisation of appropriate culture condition has been proposed for conventional formation of cultured muscle tissues, the cell types used were limited to mouse, rat, or human^13,17–19^; hence, the culture conditions for bovine muscle tissue were not clear. In this study, we found that application of electrical pulses combined with the use of fibrin-matrigel enabled contraction of all bovine muscle tissue. The electrical pulses to the tissue effectively improved their contractility, occupancy of myotubes and the proportion of matured myotubes containing striped patterns of α-actinin (Fig. 2). As the effects of electrical pulses on the bovine muscle tissue were comparable to that on muscle tissue containing myocytes of other species,^20,21^ we speculated that the mechanism underlying the maturation of myotubes via application of electrical pulses suggested in the previous studies holds good for bovine muscle tissue. For the maturation of myotubes in the bovine muscle tissue, we used 1 Hz electrical pulse, which is widely used in conventional studies.^22,23^ Some studies using muscle tissues of other species showed that the effects of myotube maturation changed with the frequency and amplitude of the electrical pulses.^20,24,25^ Therefore, we believe that further maturation of bovine muscle tissue may be facilitated by optimising the electrical pulses. Furthermore, we showed that the fibrin-matrigel mixture was more suitable for the maturation of myotubes than collagen as a culture substrate for bovine myocytes (Fig. 2), similar to that observed for maturation of myotubes in other species.^25,26^ Based on this result, we speculated that the fibrin-matrigel mixture also facilitates the formation of bovine muscle tissues via a mechanism that is identical to that underlying the maturation of myotubes in other types of muscle tissue.

One remaining issue is substitution of materials to form bovine muscle tissue for edible materials. For example, all the hydrogel we used is inedible. As there has been no edible hydrogel that can be replaced for fibrin and matrigel, as the next step toward the formation of edible contractile bovine muscle tissue, the factors in the fibrin-matrigel mixture that facilitate myotube maturation should be identified such that an edible hydrogel that meets these requirements can be developed. When looking for an edible alternative gel, it is important to consider its viscoelasticity that affects cellular motility in the gel.^27^ As the motility of myocytes in the gel is related to the rate of myotube formation, hydrogels with viscoelasticity similar to the fibrin-matrigel mixture would be essential for the edible alternative gel. Furthermore, although we use the animal serum at the present stage, it is necessary to culture the tissue using serum-free medium^28^ or a medium from algae culture^29^ for consumer acceptance and cost reduction.

Using our method, the cultured bovine muscle tissue can be easily enlarged by increasing the number of stacked modules, leading to the formation of bovine muscle tissue of standard size for steak meat. The bottlenecks in increasing the number of modules are the time-consuming steps involved in the preparation and manipulation of the modules, and difficulty in preparing sufficient bovine myocytes. The time required to manually form and stack the modules increases with the number of modules, which renders increasing the number of stacked modules difficult. To solve this issue, 3D bioprinting technology may have the potential to automate the mass production of stacked modules. In addition, many myocytes are required to prepare large numbers of modules. Therefore, by combining the automated process and mass culture of myocytes,^30,31^ the number of stacked modules will be increased, resulting in further scale-up of bovine muscle tissue.

Comparison of the mechanical properties of millimetre-thick bovine muscle tissue and commercial beef tenderloin showed that the tissue hardened with prolonged culture. As the tension of the millimetre-thick bovine muscle tissue increased with shrinkage of the tissue during culture, we believe that dense package of myotubes during the shrinkage was probably important for increasing the stiffness of the muscle tissue. In contrast, weight loss owing to heating of the commercial beef tenderloin and the muscle tissue were 40% and 90%, respectively, indicating that the density of myotubes in the muscle tissue was lower than that in the tenderloin and the water activity in the muscle tissue could be different from that in the tenderloin. On the other hand, the breaking force after heating the beef tenderloin and muscle tissue cultured for 14 days did not differ, as the water of the muscle tissue disappeared due to heating and the myotubes densely aggregated. These results may conclude that increasing the myotube density in the muscle tissue increases the tissue stiffness. As the myocyte concentration in the modules was already high (1.0 × 10^7^ cells/mL), increase in the formation rate of myotubes is important for improving the occupancy rate of myotubes in the tissue. Optimisation of electrical stimulation and hydrogel composition that were found in this paper to influence on the occupancy rate will also contribute to the improvement in the stiffness of the millimetre-thick bovine muscle tissue. In addition, as the myocytes produce extracellular matrix (ECM) such as collagen and laminin,^32^ the ECM produced in the bovine muscle tissue may contribute to the increased stiffness of the tissue.

Furthermore, in the general viable bacteria test, the microbial content of the cultured millimetre-thick bovine muscle tissue was below the detection limit, showing that microbial contamination was lower than that in commercial beef tenderloin. The sterility of the cultured tissue will promote its use as safe steak meat with long shelf life and reduce food loss.

For using the cultured bovine muscle tissue as steak meat, food characteristics such as taste and nutrient composition should be adjusted by altering the culture conditions. Analyses of amino acids and heat reaction products of cultured meat would give us important cues for the adjustment. Furthermore, the taste and nutrient of food can be controlled by food additives, as processed meat prepared by adding vitamins and heme proteins to plant-based meat is already available in the market.^33^ By combining the food additive technology with the fabrication method of bovine muscle tissue, the taste and nutrient composition of the final product can be adjusted. Moreover, the bovine muscle tissues were formed from bovine myocytes proliferated after collection of myocytes from commercial fresh beef, and not from myocytes obtained from living muscle tissues via biopsies, which suggests that the proposed bovine muscle tissue can be constructed without deviating from the existing meat supply process and also by reducing the amount of fresh beef used due to proliferation of obtained myocytes. Therefore, the proposed construction method for bovine muscle tissue of appropriate maturity and stiffness will be useful for the production of cultured steak meat of high bioethics without imposing a new burden on the livestock industry.

## Methods

### Isolation of bovine myocyte and cell culture

Bovine myocytes were obtained from commercial fresh beef isolated after slaughter (Tokyo Shibaura Zouki, Tokyo, Japan and JA ZEN-NOH Kanagawa, Kanagawa, Japan) of the Japanese black cattle (male and female, 27–30 months old). The beef was sterilised with 0.06% sodium hypochlorite (Oyalox Co. Ltd., Tokyo, Japan) to remove surface bacteria, washed twice with phosphate-buffered saline (PBS) (Roman Industries Co. Ltd., Kanagawa, Japan) and then minced with scissors or cut with thinning blades. The cell suspension was filtered using a 500-μm meshed filter and centrifuged at 400 g for 5 min after treatment with Dulbecco’s modified Eagle’s medium (DMEM) (Thermo Fisher Scientific Inc., Waltham, MA, USA) containing 0.1% collagenase (Worthington Biochemical Corp, Lakewood, NJ, USA), 1000 U/mL dispase (Wako Pure Chemical Industries Ltd., Osaka, Japan) and 50 μg/mL gentamycin sulphate (Wako Pure Chemical Industries Ltd.) at 37 °C for 1 h. Subsequently, the cells were resuspended in DMEM containing 50 μg/mL gentamycin sulphate and filtered using a 70-μm meshed filter. After centrifugation at 400 × *g* for 5 min and resuspension of cells in growth medium [DMEM containing 10% (v/v) fetal bovine serum (Thermo Fisher Scientific Inc.) and 50 μg/mL gentamycin sulphate], the cells were seeded on culture dishes. The cells were maintained in a humidified atmosphere of 5% CO_2_ at 37 °C.

To investigate population of collected cells from beef, we analysed them by flow cytometry. The collected cells were washed with PBS(−), fixed with 4% paraformaldehyde (PFA; Fujifilm Wako Pure Chemical Corporation) solution for 15 min, permeabilized with permeabilization/wash buffer (BD Biosciences) for 15 min and then incubated with rat anti-fibroblast (ER-TR7) (ACR) or mouse anti-fibroblast surface protein (1B10) (NOV) for 1 h on ice. Then, cells were washed with PBS (−) and stained with fluorescein isothiocyanate (FITC) Goat anti-rat IgG (Dako) or FITC anti-mouse IgG (Dako). Stained cells were then analysed using the FACS calibur (BD Biosciences) and Epics XL software (Beckman Coulter), and detected as fibroblasts. Subsequently, unstained cells were collected and stained with mouse anti-TGIF2 (H-36) (Santa Cruz) for 1 h on ice. After being washed with PBS (−), unstained cells were stained with FITC anti-mouse IgG and detected as myoblasts by using the FACS calibur. Similarly, unstained cells were collected and endothelial cells were detected by staining with mouse anti-Tie2/TEK (Merck Millipore), mouse VEGF-R2 (R&D Systems), or FITC anti-CD31 (Beckman Coulter). FITC anti-mouse IgG was used as second antibody. After that, satellite cells were detected by staining with APC α-7 integrin (R&D Systems).

### Construction of fibre-shaped bovine muscle tissue

Three-dimensional muscle tissue was constructed using bovine myocytes collected from beef. As parts of the culture device, anchors and a substrate were prepared based on the previous study.^34^ The anchors for immobilising muscle tissue were produced using a stereolithography apparatus (Perfactory, EnvisionTEC, Gladbeck, Germany) and were coated with 2 μm parylene using a chemical vapour deposition apparatus (Parylene Deposition System 2010, Specialty Coating Systems Inc., Indianapolis, IN, USA). The PDMS substrate to which the anchor was attached was shaped with a mould fabricated using the stereolithography apparatus. The PDMS elastomer (DuPont Toray Specialty Materials Ltd., Tokyo, Japan), mixed in the base/cross-linker ratio of 10:1, was poured into the mould. After heating it at 75 °C for 90 min, the solidified PDMS substrate was released from the mould. For sterilisation of the anchors and the substrate, they were exposed to UV light for 30 min using a steriliser apparatus (Genlantis Inc., San Diego, CA, USA). Subsequently, the substrate was coated with 2-methacryloyloxyethyl phosphorylcholine (MPC) polymer (NOF Co., Ltd., Tokyo, Japan) to prevent cell adhesion on its surface.

The culture device was prepared by setting two anchors to the substrate; the distance between the anchors was 7 mm. We prepared 2.4 mg/mL type-I collagen solution (Kurabo Industries Ltd., Osaka, Japan) or a mixture of fibrin and Matrigel [mixture of 4 mg/mL fibrinogen (Sigma-Aldrich Co. LLC., St. Louis, MO, USA), 2 U/mL thrombin (Sigma-Aldrich Co. LLC.) and 20% Matrigel (Corning Inc., Corning, NY, USA)] as the hydrogel solution. Subsequently, 30 μL hydrogel solution, collagen or fibrin-matrigel, containing bovine myocytes (5.0 × 10^7^ cells/mL) was placed on the device so as to cover the anchors at both ends of the device. After incubating the myocyte-laden hydrogel solution at 37 °C for 15 min for gelation, it was cultured in the growth medium for 2 days, followed by culturing in differentiation medium [DMEM containing 2% (v/v) horse serum (Thermo Fisher Scientific Inc.), 50 μg/mL gentamycin sulphate, 100 μM ascorbate phosphate (Wako Pure Chemical Industries Ltd.) and 100 ng/mL IGF-1 (Boster Biological Technology Ltd., Pleasanton, CA, USA)] for 12 days to form fibre-shaped bovine muscle tissues. The differentiation medium was exchanged every 2 days during the culture.

For culturing with electrical stimulation, electrical pulses (frequency: 1 Hz, duration: 2 ms, amplitude: 3 V/mm) were applied to the myocyte-laden hydrogel structures for 2 h per day from day 3 to day 14 using a C-Pace EM culture stimulation system (Ion Optix Co. Ltd., Westwood, MA, USA). The contractions of the bovine muscle tissue in response to the electrostimulation were observed using a microscope (Axio Observer D1, Carl Zeiss Co. Ltd., Jena, Germany) and their contractile displacement was measured using a motion analyser (VW-H2Ma, Keyence Corp., Osaka, Japan).

### Construction of millimetre-thick bovine muscle tissue using myocyte modules

We constructed millimetre-thick bovine muscle tissues composed of highly aligned myotubes using a modified version of a previous method^13,14^ In brief, to prepare myocyte-hydrogel modules, collagen solution (2.4 mg/mL) with myocytes (1.0 × 10^7^ cells/mL) was poured into a PDMS template formed using the same method as the PDMS substrate. The modules had holes at both ends into which pillars formed using a 3D printer (AGILISTA-3100, Keyence Corp.) were inserted. In addition, the modules had multiple slits to make striped structures in the modules (Fig. 1a). The striped structures facilitated the alignment of myocytes along the long axis of the structures, as the myocytes in the striped structure extend along its long axis.^34,35^ Two types of modules with different slit positions were stacked alternately by inserting the pillars into their holes. The immobilisation of stacked modules with pillars allowed application of cellular tension, which promoted myocyte differentiation. The stack of myocyte-laden modules was cultured in the growth medium for 1 day and in the differentiation medium for 6 days to obtain millimetre-thick bovine muscle tissues. In the case of colouring tissues, we dipped the tissues into Food Red No. 40 solution (1 mg/mL) (San-Ei Gen F.F.I., Inc., Osaka, Japan) for 30 s.

### Immunostaining

For characterisation of bovine muscle tissues using immunostaining, the tissues were washed with PBS, fixed with 4% PFA (Wako Pure Chemical Industries Ltd.), permeabilised with 0.1% Triton X-100 (Sigma-Aldrich Co. LLC) for 1 h and blocked overnight with 5% bovine serum albumin (Sigma-Aldrich Co. LLC). To visualise α-actinin, the tissues were incubated overnight with 0.1% monoclonal α-actinin antibody (Abcam plc., Cambridge, UK) at 4 °C. Subsequently, the tissues were incubated with Alexa Fluor-conjugated secondary antibodies (Thermo Fisher Scientific Inc.) at room temperature for 1 h. After immunostaining, the samples were rinsed with PBS and the cell nuclei were stained with 0.1% Hoechst 33342 (Thermo Fisher Scientific Inc.). The immunostained bovine muscle tissues were observed using a laser microscope (LSM780, Carl Zeiss Co. Ltd.).

### Morphological evaluation of bovine muscle tissue

To evaluate the maturation of the bovine muscle tissue, myotube occupancy rate and rate of formation of myotubes with α-actinin striped patterns were estimated from the fluorescent images of the immunostained tissues. For estimating the myotube occupancy rate in the tissue, the ratio of the area stained with α-actinin to the entire area of the cross-section was calculated using seven short-axial sectional images of the tissue. The rate of myotube formation with α-actinin striped patterns was calculated by counting the number of myotubes formed in the muscle tissues and the number of myotubes containing striped patterns of α-actinin from three-dimensionally stacked images of the stained tissues. FFT was applied to the fluorescent images for quantification of myotubes in the bovine muscle tissue. Greyscale pixels of FFT images were distributed in circular patterns, which reflected the myotube orientation. The radial summed pixel intensities from the FFT images showed the directional distribution of the myotubes.

### Sectional images of bovine muscle tissue

For H&E staining of the millimetre-thick bovine muscle tissue, the bovine muscle tissue was fixed with 4% PFA, dipped in 30% sucrose solution at 4 °C, and subsequently frozen in optimal cutting temperature compound (Sakura Finetek Japan Co. Ltd., Tokyo, Japan). The frozen tissue was cut with a cryostat chamber (Hyrax C25, Carl Zeiss Co. Ltd.) as 10-μm-thick sections; the sections were mounted on glass slides and stained with Mayer’s haematoxylin solution (Wako Pure Chemical Industries Ltd.) and 0.5% eosin Y ethanol solution (Wako Pure Chemical Industries Ltd.). We observed the H&E-stained sections under a microscope (M80, Leica Microsystems Ltd., Wetzlar, Germany).

### Food feature analysis of millimetre-thick bovine muscle tissue

To evaluate the food quality of the millimetre-thick bovine muscle tissue, a millimetre-thick bovine muscle tissue and commercially available beef tenderloin were cut into blocks 7 mm high and weighing 0.5 g, put in a plastic bag without air and heated at 70 °C for 1 h. After cooling for 30 min to room temperature, samples for the breaking stress test were obtained. The heating loss of the sample was calculated from the weight of the samples before and after heating. Subsequently, in this paper, we measured the breaking stress among the many ways to evaluate stiffness. For the measurement, uniaxial constant velocity indentation test was conducted for the samples using a uniaxial compression/tensile rheometer (EZ-TEST, Shimadzu Corporation, Kyoto, Japan) (measurement speed: 0.1 mm/s, plunge shaper: φ1.5 mm cylinder). In a general viable bacteria test, the millimetre-thick bovine muscle tissue and commercially available beef tenderloin were crushed in PBS to prepare sample solutions. After pouring the sample solutions aseptically into culture plates, 20 mL standard agar medium (Nissui Pharmaceutical Co. Ltd., Tokyo. Japan) was poured over the plate and carefully agitated to mix the sample solution uniformly. After incubating the plates aerobically for 48 h at 35 °C, the number of colonies on the plates was counted and expressed as colony-forming units per gram.

## Supplementary information

Supplemental video1

Supplemental video2

Supplemental video 3

Supplemental information

## Data Availability

The authors declare that all data supporting the findings of this study are available in the paper and supplementary information.
