# Supplementary material for: Formation of contractile 3D bovine muscle tissue for construction of millimetre-thick cultured steak
Source: NPJ Sci Food. 2021 Mar 2;5:6. doi: 10.1038/s41538-021-00090-7 (PMC7925560; doi:10.1038/s41538-021-00090-7)
Supplement: Supplementary file 4 — Supplemental information [file 41538_2021_90_MOESM4_ESM.pdf]

Supplementary data

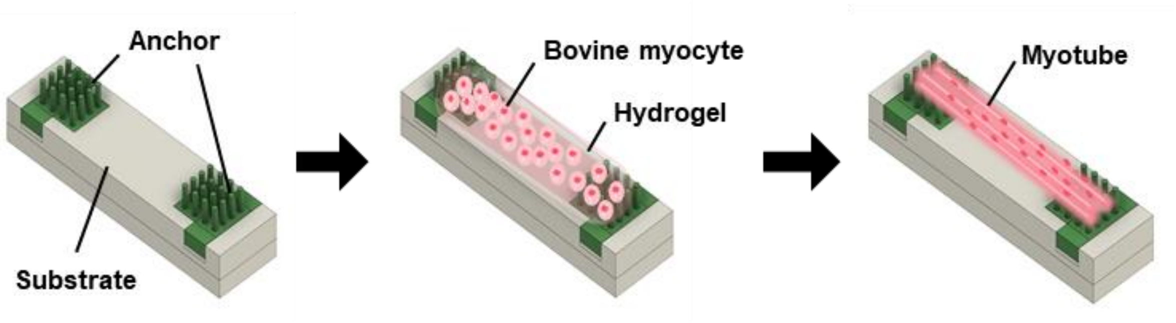

Supplementary Fig. 1. Construction process of contractile bovine muscle tissue.

Conceptual illustration of the fabrication process for a contractile bovine muscle tissue.

a

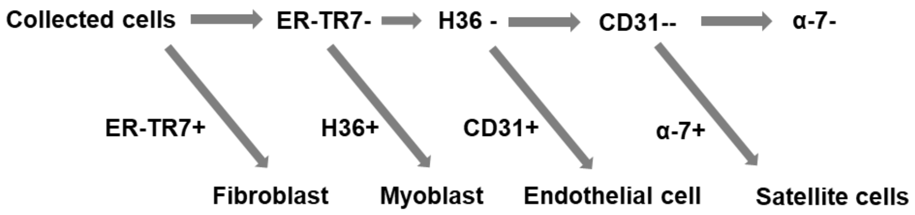

b

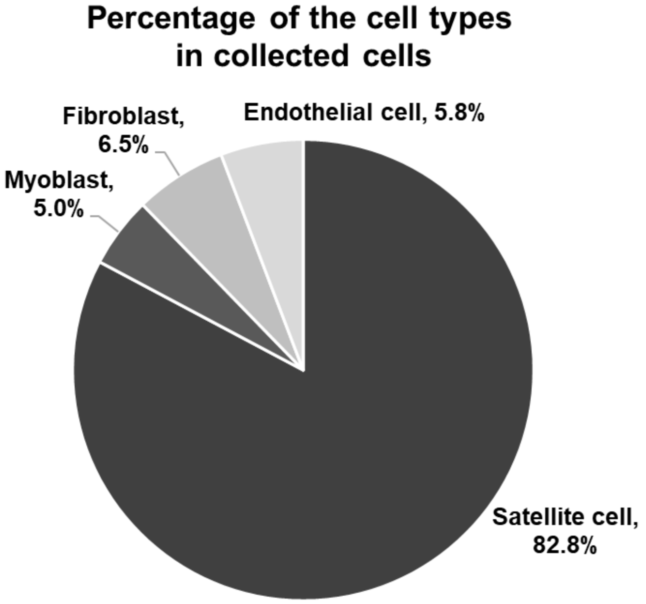

Supplementary Fig. 2. Percentage of collected cells population

a, Cell sorting method using surface marker antibody. **b**, Percentage of the bovine cell population after collected from beef.

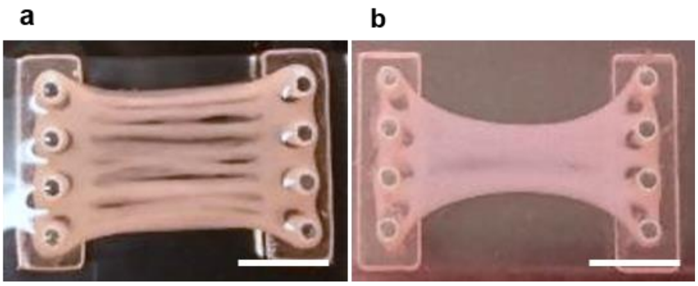

Supplementary Fig. 3. Bovine muscle tissue formed using the module assembly method. **a**, Image of 10 day-cultured muscle tissue formed with five myocyte-laden hydrogel modules (width of striped structure: 1 mm, interval between the structures: 1 mm) **b**, Image of 10 day-cultured muscle tissue formed with five myocyte-laden hydrogel modules (width of striped structure: 1 mm, interval between the structures: 1 mm).

Supplementary Video. 1. Contraction of fibrin-Matrigel-based bovine muscle tissue cultured with electrical stimulation depending on applied electrical pulses (amplitude: 3 V/mm, frequency: 1 Hz, duration: 2 ms).

Supplementary Video. 2. Contraction of collagen-based bovine muscle tissue cultured with electrical stimulation depending on the applied electrical pulses (amplitude: 3 V/mm, frequency: 1 Hz, duration: 2 ms).

Supplementary Video. 3. Manipulation of red coloured millimetre-thick bovine muscle tissue with chopsticks.
